# Asymptomatic Healthcare Worker PCR Screening during SARS-CoV-2 Omicron Surge, Germany, 2022

**DOI:** 10.3201/eid2908.230156

**Published:** 2023-08

**Authors:** Ralph Bertram, Wolfgang Hitzl, Eike Steinmann, Joerg Steinmann

**Affiliations:** Paracelsus Medical University, Nuremberg, Germany (R. Bertram, J. Steinmann);; Paracelsus Medical University, Salzburg, Austria (W. Hitzl);; Ruhr University Bochum, Bochum, Germany (E. Steinmann)

**Keywords:** COVID-19, respiratory infections, severe acute respiratory syndrome coronavirus 2, SARS-CoV-2, SARS, coronavirus disease, zoonoses, viruses, coronavirus, healthcare workers, surveillance, PCR testing, Omicron variant, Germany

## Abstract

During 2022, a total of 9,515 asymptomatic healthcare workers of a large hospital in Germany underwent SARS-CoV-2 PCR screening twice weekly. Of 398,784 saliva samples, 3,555 (0.89%) were PCR positive (median cycle threshold value 30). Early identification of infected healthcare workers can help reduce SARS-CoV-2 transmission in the hospital environment.

COVID-19, caused by the SARS-CoV-2 virus, results in acute pulmonary and extrapulmonary manifestations and frequently causes long-term sequalae (*1*). In Germany, ≈38.5 million SARS-CoV-2 infections and ≈174,000 COVID-19 deaths had been reported through May 2023 (*2*). Among those, ≈30.2 million infections and ≈47,000 deaths occurred during 2022, when SARS-CoV-2 Omicron variant dominance was accompanied by a mean hospitalization incidence of 5.87 (*2*). SARS-CoV-2 infection rates among hospitalized patients were reported to be ≈10%–15% (*3*). Healthcare workers (HCWs) also were exposed to an elevated risk of acquiring and shedding SARS-CoV-2 infections (*4*). Regular SARS-CoV-2 testing of asymptomatic HCWs has been found to reduced viral transmission to patients and coworkers (*5*). We report data from a systematic SARS-CoV-2 PCR screening program comprising >9,500 HCWs in a large hospital in Germany during 2022.

Klinikum Nürnberg is a tertiary care hospital with 2,233 beds at 2 sites in Nuremberg, Germany, and cares for ≈100,000 inpatients and ≈170,000 outpatients per year. During January–November 2022, all 9,515 hospital staff were instructed to participate in a government-mandated regular SARS-CoV-2 PCR screening program. According to federal law in Germany, participation was mandatory irrespective of the level of working exposure risk or vaccination status (Table; Appendix Table 1). 

**Table:** Characteristics and key indicators for a surveillance study among asymptomatic healthcare workers during SARS-CoV-2 Omicron surge, Germany, 2022*

Characteristic	Value
Total no. PCR tests	398,784
Median no. tests/wk (IQR)	7,559 (6,834–8,139)
Total no. PCR-positive tests	3,555
Median positivity rate, % (IQR)	0.9 (0.45–1.17)
Minimum, January 3–9	0.25
Maximum, March 14–20	1.89
Total no. HCWs tested	9,515
No. (%) infected	2,782 (29.2)
No. (%) HCWs with >2 infections	463 (4.87)
Sex, no. (%)	
M	705 (25.3)
F	2,077 (74.7)
Median age, y (IQR)	42 (30–53)
Median Ct value (IQR)	30 (27–32)
No. (%) completing immunization regimen†	8,926 (93.8)

*Ct, cycle threshold; HCW, healthcare worker. †As of March 2022 (Appendix Table 2).

Asymptomatic HCWs collected saliva samples twice weekly via self-sampling using a reliable gargling method (*6*); part-time workers collected samples less frequently. Samples were subjected to PCR testing by an external provider, and turnaround time between sampling and electronic reporting was ≈24–38 h. However, staff with acute COVID-19 symptoms were immediately PCR tested in house. Persons with PCR-verified infection were quarantined for 5–7 days and excluded from the testing program for the next 10 weeks. In November 2022, hospital staff who had no direct patient contact discontinued the screening program. 

A total of 398,784 PCR tests were performed, among which 3,555 (0.89%) were positive; 2,782 persons tested positive >1. The cumulative infection rate of all tested asymptomatic HCWs was 29.2%. We observed a minimum positivity rate (0.25%) during January 2022 and the highest numbers of positive tests in March (1.89%) and October (1.69%) 2022 (Figure, panel A). The median cycle threshold (Ct) value of all positive PCR tests was 30 (interquartile range [IQR] 27–32), suggesting that SARS-CoV-2–positive staff were detected at an early phase of infection. Asymptomatic HCWs who tested SARS-CoV-2–positive frequently had symptoms develop a few days after detection, accompanied by lower Ct values (data not shown).

**Figure:**
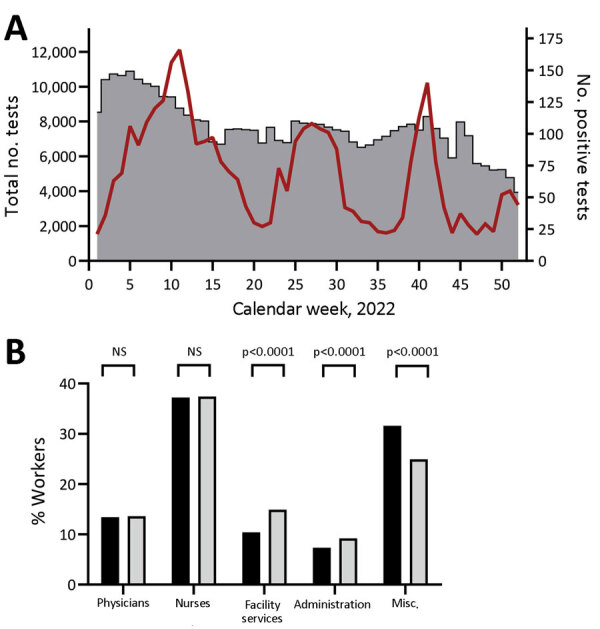
Number of tests performed and positivity rates for healthcare worker screening during SARS-CoV-2 Omicron surge, Germany, 2022. A) Total number of tests (gray bars) and number of positive tests (red line) per calendar week in 2022. B) Percentage positivity of each healthcare worker group in relation to total staff. Black bars indicate all staff; gray bars indicate the HCW groups. Misc., miscellaneous; NS, not statistically significant.

We categorized hospital staff into 5 groups: physicians, nurses, facility services, administration, and miscellaneous. Physicians constituted 13.4% of hospital staff and showed an infection rate of 13.6%. According to contingency table analysis, that rate results in a relative risk (RR) for infection of 1.02 (95% CI 0.91–1.14; p = 0.77 by Fisher exact test). Nurses accounted for 37.2% of hospital staff and exhibited an infection rate of 37.4% resulting in an RR for infection of 1.01 (95% CI 0.95–1.07; p = 0.81 by Fisher exact test). Thus, despite having the most intense contact with patients, neither of the 2 groups was significantly overrepresented in infection events (Figure, panel B; Appendix Table 1).

This 12-month SARS-CoV-2 PCR screening surveillance program of asymptomatic HCWs resulted in an average positivity rate of 0.89%. A meta-analysis of data from January–August 2020 collected by hospitals worldwide reported an average of 1.9% of asymptomatic HCWs tested PCR-positive for SARS-CoV-2 (*7*). We detected 3,555 COVID-19 cases among 2,782 (29.2%) HCWs infected >1 time. That number corresponds to results from another 12-month study in South Africa encompassing medical laboratory staff that had an overall cumulative infection rate of 25.7% (*8*). Comparisons warrant caution because of different spatiotemporal dominance of SARS-CoV-2 variants; vaccination status of HCWs, considering lower efficacy of vaccines against Omicron (*9*); and different infection control measures applied among hospitals (*10*). Furthermore, the surveillance study we report was not a randomized controlled trial, does not provide data on asymptomatic courses or rates of false positive PCR results, nor does it provide detailed information regarding seroprevalence or symptoms that developed.

The finding that physicians and nurses who were at the frontline of the COVID-19 outbreak response at Klinikum Nürnberg were not overrepresented in infection numbers speaks in favor of an efficient hygiene regimen. Besides measures such as compulsory patient screening, high-quality protective equipment, or regular ventilation, we believe that effective identification of asymptomatic HCWs in a preinfectious status might be one cornerstone of SARS-CoV-2 infection prevention in hospitals.

Appendix Additional information on asymptomatic healthcare worker PCR screening during SARS-CoV-2 Omicron surge, Germany, 2022.
