# Supplementary material for: Asymptomatic Healthcare Worker PCR Screening during SARS-CoV-2 Omicron Surge, Germany, 2022
Source: Emerg Infect Dis. 2023 Aug;29(8):1690–2. doi: 10.3201/eid2908.230156 (PMC10370853; doi:10.3201/eid2908.230156)
Supplement: Appendix — Additional information on asymptomatic healthcare worker PCR screening during SARS-CoV-2 Omicron surge, Germany, 2022. [file 23-0156-Techapp-s1.pdf]

Article DOI: https://doi.org/10.3201/eid2908.230156

EID cannot ensure accessibility for supplementary materials supplied by authors. Readers who have difficulty accessing supplementary content should contact the authors for assistance.

Asymptomatic Healthcare Worker PCR Screening during SARS-CoV-2 Omicron Surge, Germany, 2022

Appendix

Relation between Incidence Rates at Klinikum Nürnberg Hospital and in the Community

Positivity rates of the screening program were converted to the hospital healthcare worker (HCW) incidence and its trajectory was compared to that of the local incidence rates in Nuremberg and neighboring cities and counties. In the first half of 2022, the community's and the HCWs' incidences were not significantly different by independent bootstrap *t* test ($p = 0.61$), but in the second half of 2022, the incidences overall diverged significantly ($p<0.00001$), and HCWs had constantly higher rates compared with the community (Appendix Figure). Decoupling from the HCWs' and the community's incidences particularly in the second half of 2022 may be rationalized by a bona fide constant hospital-risk effect or by an increasing inaccuracy in the local incidence due to less stringent testing in the community.

Appendix Table 1. Vaccination status of among asymptomatic healthcare workers PCR screened during SARS-CoV-2 Omicron surge, Germany, 2022*

Status	No. (%)
3-Fold vaccination†	5,294 (55.6)
2-Fold vaccination†	3,002 (31.6)
1-Fold vaccination, Johnson & Johnson†	131 (1.4)
1-Fold vaccination + recovery status†	159 (1.7)
Otherwise proven sufficient immunity‡	340 (3.6)
1-Fold vaccination	33 (0.35)
Recovered without vaccination	62 (0.65)
Not vaccinated, expired recovery status, or status unknown	494 (5.2)

*As of March 22, 2022.
†Statuses considered as complete immunization.
‡Proven immunity according to German Federal Infection Protection Act (IfSG §20a).

Appendix Table 2. Statistics of infection risks for 5 healthcare worker groups screened during SARS-CoV-2 Omicron surge, Germany, 2022*

Group of HCW	% Staff	% Infections	Relative risk (95% CI)	p value
Physicians	13.4	13.6	1.02 (0.91–1.14)	0.77
Nurses	37.2	37.4	1.01 (0.95–1.07)	0.81
Facility services	10.4	14.9	1.79 (1.59–2.02)	<0.0001
Administration	7.3	9.2	1.43 (1.24–1.66)	<0.0001
Miscellaneous†	31.6	24.9	0.72 (0.67–0.77)	<0.0001

*Calculated by using Prism Version 9.5.1 (GraphPad Software Inc., https://www.graphpad.com) as contingency table analyses with χ^2 and Fisher exact test.

†Miscellaneous group includes, but is not restricted to, physiotherapists, technical assistants, teachers, and computer and natural scientists.

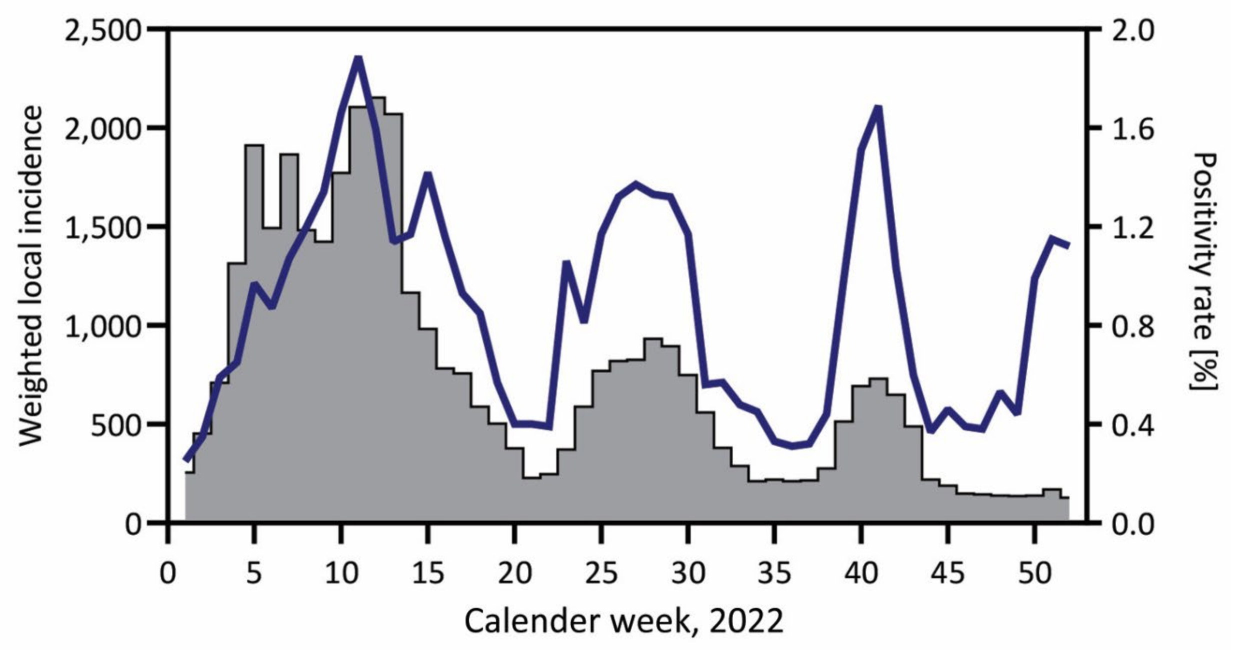

Appendix Figure. Weighted local SARS-CoV-2 incidence per calendar week of asymptomatic healthcare worker screening during SARS-CoV-2 Omicron surge, Germany, 2022. Gray indicates incidence; blue line indicates positivity rate. The weighted local incidence includes that of Nuremberg (weighted by 50%) and the incidences of the 7 surrounding cities and counties (weighted by 7.14% each) to account for different residences of healthcare workers of Klinikum Nürnberg.
